# Liver Failure in Autoimmune Hepatitis Overlap Syndrome With Primary Biliary Cholangitis: A Case Report

**DOI:** 10.7759/cureus.73574

**Published:** 2024-11-13

**Authors:** Marta Batista, Patricia Brito, Pedro Miranda, Sandra Barbosa, Jorge Cotter

**Affiliations:** 1 Internal Medicine, Hospital Senhora da Oliveira, Guimarães, PRT

**Keywords:** acute liver failure (alf), autoimmune hepatitis, overlap syndrome, primary biliary cholangitis, ursodeoxycholic acid

## Abstract

Autoimmune hepatitis (AIH) is a complex and long-term liver condition, primarily affecting women, and is marked by high levels of serum gamma globulins, the presence of circulating autoantibodies, and a genetic association. The disease can manifest in a variety of ways, ranging from mild or no symptoms to severe acute liver inflammation. AIH is often associated with other autoimmune disorders, such as primary biliary cholangitis (PBC) and autoimmune thyroiditis. The progression of the disease can lead to cirrhosis or liver failure if not treated with immunosuppressive therapy. Overlap syndromes, such as AIH with PBC, further complicate both diagnosis and management due to the lack of standardized treatment protocols. The rarity of AIH and the absence of large-scale, randomized clinical trials significantly limit current treatment strategies, underscoring the need for ongoing research to improve therapeutic approaches. We report on a 54-year-old female patient who was admitted with acute hepatitis that rapidly progressed to liver failure. After ruling out other causes, corticosteroid treatment was started based on a suspected diagnosis of AIH. Liver biopsy results supported the diagnosis of an overlap syndrome involving AIH and PBC.

## Introduction

Autoimmune hepatitis (AIH) is a chronic liver condition predominantly affecting women. It is characterized by elevated serum gamma globulin levels and the presence of circulating autoantibodies, even in the absence of cirrhosis. Histologically defined by interface hepatitis, AIH generally responds well to immunosuppressive therapy. However, without timely treatment, it may progress to cirrhosis, liver failure, and potentially fatal outcomes [1-4].

The prevalence of AIH is relatively low, estimated at 16-18 cases per 100,000 individuals in Europe [1,5]. Clinical presentations vary widely, influenced by factors such as ethnic background, genetic predispositions, environmental influences, and pharmacogenomic interactions. Socioeconomic factors may also contribute to these variations [1,6]. AIH can range from asymptomatic cases to acute and severe manifestations and, in some cases, may progress to fulminant hepatitis [1].

AIH is classified into subtypes based on autoantibody profiles, with AIH-1 being the most prevalent. AIH-1 is characterized by antinuclear antibodies (ANA), smooth muscle antibodies (SMAs), or anti-soluble liver antigen/liver-pancreas (SLA/LP) antibodies and is linked to HLA-DR3, DR4, and DR13. Additional subtypes, AIH-2 and possibly AIH-3, display unique antibody profiles, highlighting the disease's complexity and the critical need for timely diagnosis and management [1,7].

Overlap syndromes, where AIH coexists with other autoimmune liver diseases such as primary biliary cholangitis (PBC), occur in about 8-10% of adults diagnosed with either condition. Diagnosis often relies on the "Paris criteria," which requires at least two of the three primary diagnostic features for each disease. For PBC, these include elevated alkaline phosphatase (ALP) level greater than twice the upper normal limit (ULN) or gamma-glutamyl transpeptidase exceeding five times the ULN, presence of antimitochondrial antibodies (AMA), and liver biopsy revealing florid bile duct lesions. For AIH, the criteria are alanine aminotransferase (ALT) levels five times the ULN, serum IgG levels at least twice the ULN or the presence of SMA, and liver biopsy demonstrating moderate to severe periportal or periseptal lymphocytic piecemeal necrosis. These conditions may require specialized treatment approaches, particularly if patients do not respond well to standard therapies like ursodeoxycholic acid (UDCA) alone [1,7,8].

Due to the rarity and complexity of AIH-PBC overlap syndrome, standardized treatment protocols are not established. Studies show variable outcomes with combinations of UDCA and immunosuppressive agents like corticosteroids or thiopurines. For those unresponsive to initial therapy, alternative options such as azathioprine or mycophenolate mofetil may be considered [7].

## Case presentation

A 54-year-old female presented to the emergency department with diffuse abdominal pain, most pronounced in the right hypochondrium. Her symptoms included anorexia, nausea, vomiting, jaundice, and dark urine, which had been progressively worsening over the past three weeks, with an acute exacerbation in the last three days. In addition, she reported experiencing worsening nocturnal sweats, arthralgias, myalgias, and aquagenic pruritus over the past month.

The patient denied any use of alcohol, illicit drugs, herbal supplements, teas, wild mushrooms, raw eggs, or unpasteurized products. She reported no epidemiological risks, including recent surgeries, previous hospitalizations, known drug allergies, high-risk sexual behavior, recent travel, or contact with animals.

On physical examination, the patient was hemodynamically stable, afebrile, and conscious with intact orientation and no neurological deficits. Jaundice was present, along with tenderness on deep palpation in the right and left hypochondrium, as well as the left iliac fossa. No palpable masses or nodules were detected, and joint examination revealed no abnormalities. Cardiac and pulmonary auscultation were unremarkable. In addition, the patient exhibited palmar erythema, without other stigmata of chronic liver disease.

Laboratory tests revealed mild thrombocytopenia, cholestasis, hypoalbuminemia, coagulopathy, and an elevated C-reactive protein level. These findings are summarized in Table 1.

**Table 1:** Laboratory investigations Abbreviations: INR, international normalized ratio; NM, not measured

Analyte	Patient value				Reference range
	On presentation	Day 2	Day 10	Discharge	
Hemoglobin (g/dL)	13.2	9.9	9.1	9.9	12-16
Hematocrit (%)	39.1	29.7	30.7	29.9	40-50
Mean corpuscular volume (fL)	98.2	100.3	97.4	104.9	83-103
Mean corpuscular hemoglobin (pg)	33.2	33.4	28.9	34.7	28-34
Mean corpuscular hemoglobin concentration (g/dL)	33.8	33.3	29.7	33.1	32-36
Leukocytes (x10^3^/µL)	8.8	1.9	1.7	8.8	4.8-10.8
Neutrophils (%)	89%	59.2%	39.3%	76.6%	38-70
Lymphocytes (%)	5.8%	22.2%	26%	13.6%	20-40
Basophils (%)	0.1%	0%	0.6%	0.1%	0-1
Eosinophils (%)	0.5%	9.3%	11%	1.8%	1-6
Monocytes (%)	4.1%	8.8%	22.5%	7.2%	2-10
Platelets (x10^3^/µL)	148	93	89	101	150-300
C-reactive protein (mg/L)	48.5	34.6	29.1	7.0	<3
Total bilirubin (mg/dL)	11.03	10.93	23.75	6.42	0.3-1.2
Direct bilirubin (mg/dL)	8.50	7.86	17.06	5.14	0.0-0.3
Aspartate aminotransferase (IU/L)	1434	1063	1431	183	12-40
Alanine aminotransferase (IU/L)	1433	1057	784	367	7-40
Gamma-glutamyl transferase (IU/L)	178	145	136	191	0-73
Lactic dehydrogenase (IU/L)	471	353	405	198	120-246
Alkaline phosphatase (IU/L)	153	114	173	135	46-116
Albumin (g/dL)	3.1	2.8	2.6	3.0	3.4-5
INR	1.8	1.7	1.8	1.4	
Factor V (%)	NM	NM	65%	NM	62-139
Ammonia (µg/dL)	NM	NM	102	NM	19-54

An abdominal ultrasound was performed given the clinical presentation, revealing the following findings: A dysmorphic liver with slight hypertrophy of the left lobe relative to the right, irregular contours, and finely heterogeneous parenchyma. There was mild dilation of the intrahepatic bile ducts without significant dilation of the main bile duct. In addition, increased echogenicity of the portal triads was noted, potentially indicative of periportal edema. The gallbladder displayed normal dimensions and wall thickness, with no signs of acute cholecystitis. The spleen was of normal size with a homogeneous parenchyma.

The patient was admitted for further evaluation of acute hepatitis of unknown etiology. During hospitalization, an etiological investigation was conducted as detailed in Table 2, which included an autoimmune workup in Table 3. An abdominal Doppler ultrasound was performed, excluding portal vein thrombosis. In addition, an abdominal MRI and magnetic resonance cholangiopancreatography were performed, which excluded neoplastic processes. Upper gastrointestinal endoscopy revealed congested mucosa with a mosaic pattern in the gastric fundus and body, consistent with mild portal hypertensive gastropathy. In addition, areas of erythema were noted in the antral mucosa, with no other significant findings. The patient underwent an ophthalmologic evaluation, which ruled out the presence of Kayser-Fleischer rings. A 24-hour urinary copper excretion was measured at 112 µg/24 hours. On the second day of hospitalization, the patient developed pancytopenia, as shown in Table 1. A peripheral blood smear demonstrated anisocytosis and anisocromia, with no evidence of platelet aggregates. A bone marrow biopsy was performed, revealing no abnormalities.

**Table 2:** Additional laboratory investigations for acute hepatitis

Analyte		Reference range
Alcohol (mg/dL)	<3	>10
Serum iron (µg/dL)	80	50-170
Total iron-binding capacity (TIBC) (µg/dL)	259	250-425
Ferritin (ng/mL)	1357.50	10-291
Transferrin saturation	31%	
Serum folate (ng/mL)	14.6	> 5.38
Vitamin B12 (pg/mL)	1402	211-911
Angiotensin-converting enzyme (IU/L)	49	35-90
Alpha-fetoprotein (ng/mL)	21.1	<8.1
Ceruloplasmin (mg/dL)	20.1	25-63
Alpha-1 antitrypsin (mg/dL)	175	78-200
Haptoglobin (mg/dL)	<7.1	16-200
Complement C3 (mg/dL)	92.5	82-170
Complement C4 (mg/dL)	15	12-36
Total hemolytic complement activity (CH50) (mg/dL)	15	12-36
IgM (mg/dL)	84.2	50-350
IgA (mg/dL)	261.2	40-350
IgG (mg/dL)	2133	650-1600
Cytomegalovirus IgM	Negative	
Cytomegalovirus IgG	Positive	
Epstein-Barr virus IgM	Negative	
Epstein-Barr virus IgG	Positive	
Herpes simplex virus 1 IgM	Negative	
Herpes simplex virus 1 IgG	Positive	
Herpes simplex virus 2 IgM	Negative	
Herpes simplex virus 2 IgG	Positive	
Toxoplasmosis IgM	Negative	
Toxoplasmosis IgG	Positive	
Parvovirus B19 IgM/IgG	Negative	
Varicella zoster virus IgM	Negative	
Varicella zoster virus IgG	Negative	
Syphilis	Negative	
Hepatitis A IgM	Negative	
Hepatitis A IgG	Positive	
Hepatitis B surface antigen (HBsAg)	Negative	
Hepatitis B surface antibody (anti-HBs)	Negative	
Hepatitis B core antibody (anti-HBc) IgM	Negative	
Hepatitis C antibody (Anti-HCV) IgG	Negative	
HIV-1/2 antigen/antibody	Negative	
Hepatitis E IgM	Negative	
Hepatitis E IgG	Negative	
Interferon gamma release assay (IGRA)	Negative	
Antibody anti-Brucella IgM/IgG	Negative	
Antibody anti-Borrelia IgM/IgG	Negative	
Leptospira IgM	Negative	

During hospitalization, the patient experienced fulminant clinical deterioration, culminating in acute liver failure on the 10th day of admission. At that time, infectious, metabolic, ischemic, and neoplastic etiologies had been excluded based on the results obtained, and only part of the immunologic study was available, which showed elevated IgG levels and positive PR3-anti-neutrophil cytoplasmic antibody (ANCA), with a C-ANCA pattern. The remaining results were pending, and the case was discussed with the transplantation center. During the discussion, given the exclusion of other etiologies and the clinical presentation, the findings were highly suggestive of AIH. Consequently, corticosteroid therapy was initiated at a dose of 60 mg/day (<1 mg/kg/day), and the case was notified to the transplant center, which could be contacted at any time.

On the 13th day of hospitalization (the third day of corticosteroid therapy), the patient's coagulopathy resolved, and a liver biopsy was performed without complications. The autoimmune study subsequently revealed abnormalities as detailed in Table 3, including positive antinuclear antibody (ANA) with an AC-15 cytoplasmic fibrillar linear pattern and co-positivity for anti-smooth muscle antibody (ASMA) and anti-AML-F antibody.

**Table 3:** Autoimmune study

Analyte		Reference range
Antinuclear antibody	Positive 1:160 AC-15 cytoplasmic fibrillar linear	<1:80
Anti-smooth muscle antibody (ASMA)	Positive 1:40	<1:40
Anti-AML-F antibody	Positive	
Anti-neutrophil cytoplasmic antibody (ANCA)	Positive 1:40 C-ANCA pattern	<1:20
ANCA PR3 (IU/L)	12.5	<5
Anti-MPO antibodies (IU/mL)	<1.0	<6
Anti-mitochondrial antibodies (AMA)	Negative	
Anti-liver kidney microsomal (LKM) antibodies	Negative	
Anti-soluble liver antigen (SLA) antibodies	Negative	
Anti-liver cytosol type 1 (LC1) antibodies	Negative	
Gp210	Negative	
Sp100	Negative	

The patient showed both clinical and biochemical improvement, with resolution of acute liver failure following corticosteroid therapy at 60 mg/day.

The liver biopsy revealed the following findings: preservation of the hepatic parenchymal trabecular architecture, moderate portal fibrosis without fibrous septa in the parenchyma, and a portal infiltrate predominantly composed of mononuclear cells with some plasma cells, accompanied by moderate interface necrosis. Mild necro-inflammatory intralobular lesions and pseudo-rosette arrangements of some hepatocytes were observed, along with ductular reaction, bile duct hyperplasia, and bile duct destruction. No megamitochondria or Mallory bodies were observed, and there was no evidence of steatosis, siderosis, or cholestasis.

According to the Paris criteria, the patient was diagnosed with overlap syndrome of primary biliary cholangitis and AIH. Consequently, ursodeoxycholic acid was added at a dose of 500 mg twice daily. The patient has been followed up and has achieved complete resolution of cytocolestasis, with no disease flare-ups reported to date.

## Discussion

In cases of acute hepatitis, it is crucial to perform an etiological study of acute hepatitis to guide treatment and prevent the progression to acute liver failure [1,4]. In this instance, the patient showed no epidemiological context indicative of a viral or toxic etiology. Nevertheless, it remains important to rule out these causes, alongside obstructive, neoplastic, metabolic, and ischemic origins [1,4].

After excluding all other potential causes and despite incomplete immunological study results, the patient demonstrated elevated IgG levels and was positive for ANCA-PR3. AIH, predominantly observed in women, is commonly associated with elevated gammaglobulin levels [1,2,4]. Therefore, the combination of hypergammaglobulinemia, female gender, and the exclusion of metabolic, infectious, ischemic, and neoplastic conditions strongly suggested an autoimmune etiology.

When there is an unfavorable progression to acute liver failure, a multidisciplinary approach and early referral to a transplantation center are essential [1]. This case was also discussed with the transplant center and planned to proceed with transplantation if the patient's condition deteriorated.

AIH has a highly heterogeneous clinical course, ranging from subclinical presentations to acute forms, although it rarely progresses to fulminant acute liver failure [1-4]. In this patient, such progression occurred, and when a liver biopsy was planned, she developed coagulopathy and a general deterioration in her condition, leading to a postponement of the procedure.

In these cases, performing a liver biopsy is essential and should be undertaken at the initial presentation, as it helps establish an early diagnosis [1,3]. In patients with coagulopathy, there is an increased risk of bleeding; therefore, a transjugular liver biopsy should be considered. In selected cases, laparoscopic biopsy may be contemplated, although it is a more invasive procedure [1,3]. However, not all centers have immediate access to this technique. Consequently, due to the patient’s deterioration, the biopsy could only be performed after the initiation of corticosteroid therapy. Once there was clinical improvement and resolution of the coagulopathy, an ultrasound-guided biopsy was performed.

In this case, despite initiating corticosteroid therapy three days prior to the biopsy, histological analysis revealed several findings suggestive of AIH, including interface hepatitis, a portal infiltrate predominantly composed of mononuclear cells with some plasma cells, and hepatocellular rosette formation. The biopsy also showed features indicative of primary biliary cholangitis (PBC), including ductular reaction, bile duct hyperplasia, and bile duct destruction. 

The diagnosis of an AIH-PBC overlap syndrome can be established based on the "Paris criteria," which require at least two of the three primary diagnostic features for each disease. For PBC, these features include elevated alkaline phosphatase (ALP) levels greater than twice the upper limit of normal (ULN) or gamma-glutamyl transpeptidase (GGT) exceeding five times the ULN; the presence of antimitochondrial antibodies (AMA); and liver biopsy revealing florid bile duct lesions. For AIH, the criteria are alanine aminotransferase (ALT) levels exceeding five times the ULN; serum IgG levels at least twice the ULN or the presence of smooth muscle antibodies (SMA); and liver histology demonstrating moderate to severe periportal or periseptal lymphocytic piecemeal necrosis [1,7-9]. According to these criteria, the patient was diagnosed with an overlap syndrome.

When AIH is suspected in the context of cholestasis, an overlap syndrome should be considered, as was observed in this case [1,7-9]. As described in the literature, patients with overlap syndromes may have other associated autoimmune diseases [1-4]. Consequently, further investigation was conducted, leading to the diagnosis of autoimmune thyroiditis with associated hypothyroidism.

Due to the rarity and complexity of AIH-PBC overlap syndrome, standardized treatment protocols are not established [7,9]. Studies show variable outcomes with combinations of UDCA and immunosuppressive agents. For those unresponsive to initial therapy, alternative options such as azathioprine or mycophenolate mofetil may be considered [7-9].

Studies have shown variable outcomes when combining UDCA with immunosuppressive agents. For patients unresponsive to initial therapy, which occurs especially in those already with fibrosis, alternative options such as azathioprine or mycophenolate mofetil may be considered [7-9].

The patient was started on a corticosteroid regimen with a gradual taper, and azathioprine was introduced progressively according to the European Association for the Study of the Liver (EASL) Clinical Practice Guidelines: Autoimmune Hepatitis [1,3]. Ursodeoxycholic acid was also added at a dose of 13-15 mg/kg per day [9].

When initiating azathioprine therapy, it is important to assess the activity of thiopurine methyltransferase (TPMT). This enzyme is crucial for the metabolism of azathioprine and can help predict the risk of toxicity [1]. The patient had normal thiopurine methyltransferase (TPMT) levels and showed a good response to therapy without new exacerbations.

However, after approximately six months of azathioprine treatment, she developed dyspnea accompanied by ground-glass opacities on imaging studies. An etiological workup ruled out other causes, and her therapy was switched to mycophenolate mofetil, resulting in complete resolution of the pulmonary condition. It has been reported that up to 25% of patients with AIH develop side effects from azathioprine, resulting in the discontinuation of the medication in about 10% of cases [1].

Lastly, in these cases, it is also important to monitor the risk of developing hepatocellular carcinoma and to be vigilant about the inherent risks of immunosuppression, such as infections.

## Conclusions

AIH presents with a heterogeneous clinical course and rarely progresses to acute liver failure. Clinically, it can range from being asymptomatic to manifesting various nonspecific symptoms such as fatigue, weight loss, abdominal pain, and jaundice. AIH may also be associated with other autoimmune diseases, such as primary biliary cholangitis and autoimmune thyroiditis, among others. This case highlights the importance of differential diagnosis and the need for early liver biopsy to complete and clarify the etiological study in complex cases, as well as to guide therapeutic decisions. A multidisciplinary and prompt approach is essential to prevent the development of acute liver failure and to ensure early referral to a liver transplantation center.
